# Hippo and *rassf1a* Pathways: A Growing Affair

**DOI:** 10.1155/2012/307628

**Published:** 2012-07-05

**Authors:** Francesca Fausti, Silvia Di Agostino, Andrea Sacconi, Sabrina Strano, Giovanni Blandino

**Affiliations:** ^1^Molecular Chemoprevention Group, Molecular Medicine Area, Regina Elena Cancer Institute, Via Elio Chianesi 53, 00143 Rome, Italy; ^2^Translational Oncogenomic Unit, Molecular Medicine Area, Regina Elena Cancer Institute, Via Elio Chianesi 53, 00143 Rome, Italy

## Abstract

First discovered in *Drosophila*, the Hippo pathway regulates the size and shape of organ development. Its discovery and study have helped to address longstanding questions in developmental biology. Central to this pathway is a kinase cascade leading from the tumor suppressor Hippo (Mst1 and Mst2 in mammals) to the Yki protein (YAP and TAZ in mammals), a transcriptional coactivator of target genes involved in cell proliferation, survival, and apoptosis. A dysfunction of the Hippo pathway activity is frequently detected in human cancers. Recent studies have highlighted that the Hippo pathway may play an important role in tissue homoeostasis through the regulation of stem cells, cell differentiation, and tissue regeneration. Recently, the impact of RASSF proteins on Hippo signaling potentiating its proapoptotic activity has been addressed, thus, providing further evidence for Hippo's key role in mammalian tumorigenesis as well as other important diseases.

## 1. Introduction

**Figure 1:**
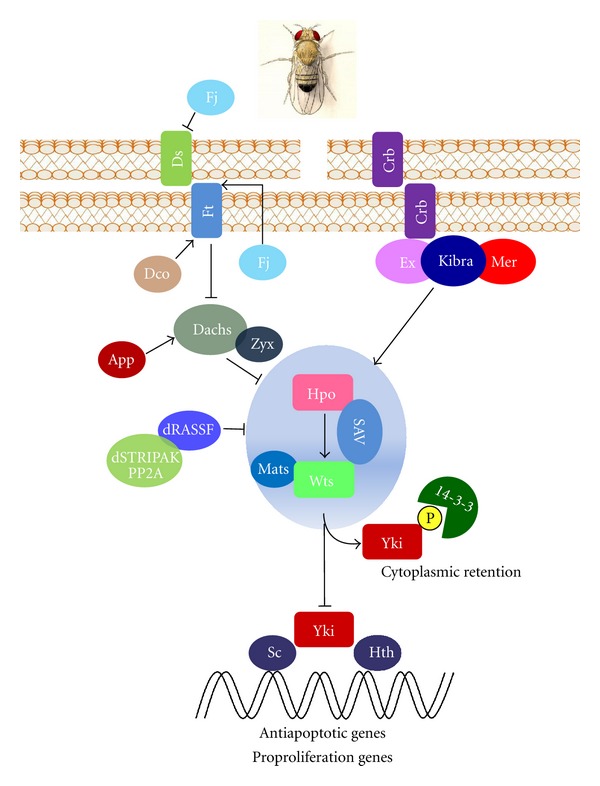
“Hpo signaling pathway in *Drosophila.*” Schematic representation of Hippo kinases cascade and of its modulation by apical transmenbrame protein complexes.

**Figure 2:**
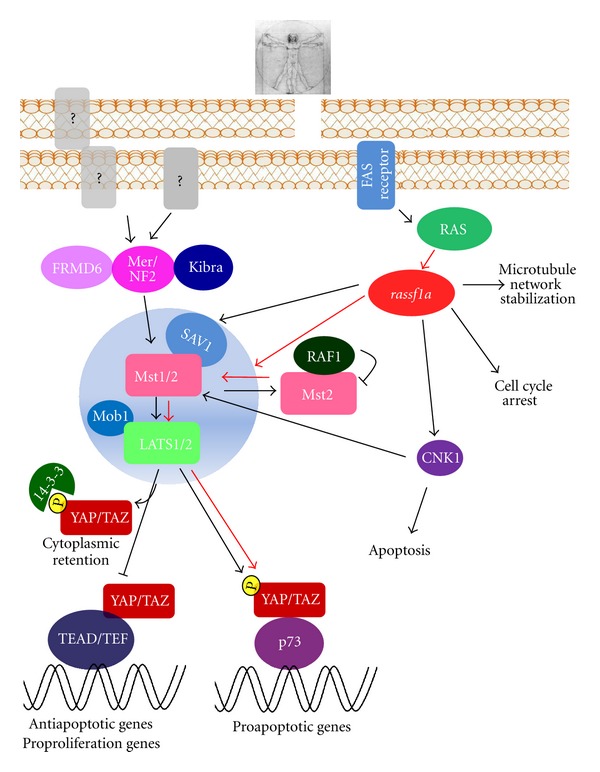
“Hpo signaling pathway in Mammals and the cross-talk with RASFF1A signaling.” Schematic representation of mammalian Hippo kinases cascade and interconnections between Hippo pathway and *rassf1a* protein signal. Red lines indicate the impact of *rassf1a* signaling in modulating activity of Hpo pathway components.

The Hippo pathway is a signaling pathway that regulates cell growth and cell death. It was discovered in *Drosophila melanogaster* as a pathway controlling organ size and of which mutations lead to tumorigenesis. This pathway is highly conserved, and its activation or repression could lead to the following most extreme outcomes: proliferation/transformation and death/tumor suppression. The Hippo pathway cross-talks with other signaling players such as Notch, Wnt, and Sonic hedgehog (Shh). It influences several biological events, and its dysfunction may possibly lie behind many human cancers. In this review, we discuss the complex data reported about *Drosophila* to date (schematic representation in Figure 1) and the human Hippo (schematic representation in Figure 2) pathways focusing on the relationship between the tumor suppression *rassf* protein family and the Hippo-like pathway in humans [1, 2].

## 2. The Hippo Signaling Network in *Drosophila*

*Drosophila* imaginal discs have facilitated molecular dissecting of signaling pathways controlling organ size during development. These imaginal discs allow to screen how organs grow several folds larger before differentiating into adult organs after proliferation in larval stages. By using the genetic analysis in *Drosophila*, Robin W. Justice and colleagues were the first to describe that loss of Wts (Warts), which encodes a kinase of Nuclear Dbf-2-related (NDR) family, results in a *Drosophila *phenotype characterized by tissue overgrowth [3]. Several years later many components of this pathway were characterized. Four tumor suppressors called Hippo (Hpo), Warts (Wts), Salvador (Sav), and Mats were established. These suppressors constitute the core linear kinase cassette of Hippo/Warts pathway whose products can affect proliferation without increasing apoptosis susceptibility [3–6] (Figure 1). Subsequent genetic screens identified at least seven additional tumor suppressors whose biological functions converge on Hpo and/or Wts: the FERM domain proteins Merlin (Mer) and Expanded (Ex) [7–10], the protocadherins Fat (Ft) [11–14] and Dachsous (Ds) [15, 16], the CK1 family kinase Disc overgrown (Dco) [17, 18], the WW and C2 domain-containing protein Kibra [19–21], and the apical transmembrane protein Crumbs (Crb) [22–24]. All of these suppressors converge and act through a common downstream component, the transcriptional co-activator protein Yorkie (Yki) [25] (Figure 1). The mechanisms by which these upstream regulators signal towards the final player Yorkie are complex and are still focus of investigation. A great deal of evidence suggests that they work in a combinatorial or synergistic manner to regulate Hippo kinase activity.

### 2.1. The Apical Protein Complex: Kibra, Expanded, and Merlin

The molecular link between upstream regulators and the core complex has not yet been clarified in mammals nor in *Drosophila.* In 2006, Hamaratoglu and collaborators proposed Mer (Merlin) and Ex (Expanded) as potential upstream regulators of the Hippo pathway [9], proteins which contain a FERM (4.1/ezrin/radixin/moesin) domain. Both proteins are considered tumor suppressors which cooperate to control organ growth. Their function seems to be partially redundant. In fact, while single mutation of each gene results in increased tissue growth, mutations in both genes give rise to a more strongly affected phenotype [9, 10]. Kibra, a third component of this apical complex, has recently been found. This protein possesses a WW domain which facilitates the interaction with other members of the Hippo pathway, such as Wts. It further interacts with a C2 domain that consists of a phospholipid-binding motif through which Kibra is believed to potentiate its membrane association [19–21]. WW domains are 35–40 amino acid protein–protein interaction domains that are characterized by a pair of conserved Trp residues, which generally interact with Pro-rich sequence motifs [26]. WW domain-Pro motif interactions appear to be particularly common in the Hpo pathway. Three core components of Hpo signaling (Yki, Kibra, and Sav) contain WW domains, whereas three other components (Wts, Ex, and Hpo) hold PPxY motifs (reviewed in [27, 28]). While the formation of a ternary complex between Kibra, Ex, and Mer was observed, each protein was seen to localize to cellular membranes independently. Furthermore, it has been published that the Kibra-Mer-Ex complex is physically involved with the Hpo-Sav, constituting an apical protein complex required for associating the Hpo pathway to the cellular membranes [20, 21]. Studies on the Ex localization and function have led to the discovery of another important upstream regulator protein of Hpo, Crb (Crumbs) [22–24]. Crb is a transmembrane protein which normally localizes to the subapical membrane of epithelial cells that is responsible together with other apical complexes in *Drosophila* for organizing apical-basal polarity [29]. Crb binds to Ex through a short intracellular domain including a juxtamembrane FERM-binding motif (FBM). The FBM domain of Crb interacts with the FERM domain of Ex. This type of binding is necessary for Ex apical localization and stability. Furthermore, it has been published that Crb also works with Mer and Kibra [23]. The loss of Crb expression was shown to further determine a phenotype characterized by overgrowth, possibly to a lesser degree compared to the other members of Hpo signaling described until now [22–24]. Not long ago, this protein was proposed to have had an important function as a transmembrane receptor recognizing cell-cell contacts through Crb-Crb binding domains [22].

### 2.2. The Upstream Regulator: Transmembrane Protein Fat

The atypical cadherin FAT (Ft) was the first transmembrane protein shown to affect Hippo signaling. Fat is the first tumor suppressor gene isolated in *Drosophila*. In fact, the complete knock-out of the FAT protein induces death in *Drosophila *larvae with overgrown imaginal discs [11]. As previously mentioned, FAT is a large transmembrane protein, constitutively cleaved by unknown proteases. It contains 34 cadherin repeats in its extracellular domain, functioning as a receptor for Hippo signaling [12–14] as well as for planar cell polarity (PCP) [30, 31]. PCP is a mechanism through which cells orient themselves orthogonally to the apical-basal axis, as observed in the wing hairs of *Drosophila*, and the sensory hair cells in the inner ear of mouse. Notably, the mechanism by which FAT regulates Hippo signaling is different from the branch involving the ternary complex Ex-Mer-Kibra. Many lines of evidence suggest that the principal mechanism exerted by FAT is on the Wts function [18, 32]. Thus, FAT-Hpo signaling is genetically distinguishable, involved in Hippo pathway regulation of imaginal discs and neuroepithelial tissue, but not in other tissues such as ovarian tissue [14, 33, 34]. Many genes were reported to take part in this parallel mechanism together with FAT. First, Dachsous (Ds), an atypical cadherin which binds to FAT [15, 16]. FAT is regulated by an expression gradient of Ds [35, 36]. Four-jointed (Fj) is a kinase that typically localizes to the Golgi subcellular compartment and that phosphorylates the cadherin domains of FAT and Ds to mediate binding between these two proteins [37]. Another kinase responsible for FAT phosphorylation in its cytoplasmatic segment is a Casein I kinase, termed Discs overgrown (Dco) [17, 18]. The effective key mediator of FAT in the Hippo pathway seems to be Dachs, an unconventional myosin which antagonizes FAT, and whose activity is influenced by Approximated (App) [17]. App, in fact, antagonizes FAT signaling by modulating Dasch expression [38]. Another protein identified recently linked to the FAT branch in Hippo signaling is the LIM-domain protein Zyx102. It has been found to directly affect the core kinases of the Hippo pathway [39]. All of these components described above seem to be responsible for linking Hippo to extracellular stimuli [40].

Another so called “scaffold” protein that has been identified as a regulator of Hpo is called *Drosophila* *rassf* (*drassf*). This protein like its mammalian counterpart *rassf* can bind to Hpo through a conserved SARAH domain. But unlike in mammals, it hampers Hpo activity by competing with SAV to bind to Hpo [41] and by recruiting a Hpo-inactivating PP2A complex (dSTRIPAK) [42], thus showing a positive regulation of growth. Interestingly, Grzeschik and collaborators showed that the depletion of the *Drosophila *neoplastic tumor suppressor Lethal giant larvae (Lgl), which controls apical-basal cell polarity and proliferation, leads to upregulation of the Hippo pathway target Yki through a decreased phosphorylation and consecutively overproliferation of developing eyes, without affecting apical-basal polarity [43]. This mechanism is brought about by cellular mislocalization of Hpo and *rassf*. These both colocalize basolaterally leading to the deregulation of the Hippo kinase cascade, thereby preventing phosphorylation and inactivation of Yki. This concurs with data previously discussed wherein *rassf* is able to bind to Hpo precluding its interaction with SAV [41]. 

### 2.3. The Key Effectors of Growth Control: Hippo, Warts, Salvador, and Yorkie

Warts is crucial in the phosphorylation-dependent regulation of Yki [25, 44, 45]. Warts (Wts) encodes a Ser/Thr kinase of Nuclear Dbf-2-related (NDR) family. The activity of Warts is controlled through a series of phosphorylation events. Warts is directly phosphorylated by Hippo (Hpo), a member of the Sterile-20 family of Ser/Thr kinases, in a reaction that is facilitated by the Salvador protein [4, 5]. The fly protein Hippo (Hpo) is the first mediator of this pathway characterized by a kinase cascade. Wu and collaborators identified Hpo through analysing the phenotype of *Drosophila* Hpo mutants. Hpo is a kinase protein that regulates cell proliferation as well as apoptosis in *Drosophila. *In addition, it interacts, phosphorylates, and is activated by the WW domain-containing protein Salvador. Salvador (Sav) was described as a tumor suppressor gene, whose loss caused tissue overgrowth, similar to Wts loss of function. Tapon and collaborators were the first to observe, in 2002, that loss of Sav or Wts was strictly associated with increased expression of *cyc* *e*, a cell cycle progression regulator and *diap1*, an apoptosis inhibitor, thus, confirming these that two proteins' very important role in coordinating these two cellular processes [4]. Similar to Sav function on Hpo, Mats' role (Mob as tumor suppressor) which also belongs to the NDR family, as well as its kinase-like behavior binding to and potentiating Wts intrinsic activity, was described in 2005 [6]. Thus, Sav and Mats action as adaptor proteins, often termed scaffold proteins, both serve to potentiate Hippo signaling. Interestingly, it was also reported that Mats is a Hpo substrate. The latter phosphorylates Mats increasing its affinity for Wts binding, thus inducing potentiation of Wts kinase activity [46].

The downstream key regulator of Hpo signaling is Yorkie (Yki). It was identified in a yeast two-hybrid screen for Wts-binding protein, which is the final step in the Hippo pathway, driving its transcriptional regulation [25]. Yki is not a direct transcriptional factor because it does not possess its own consensus DNA-binding motif but is known as a potent transcriptional co-activator by cooperating with different DNA-binding proteins. Wts directly phosphorylates Yki at Ser 168, thus creating a binding site for 14-3-3 proteins which sequester Yki in the cytoplasm and prevent its nuclear import [44, 45]. In actual fact, the loss of Hippo signaling as well as mutations in 14-3-3 binding site for Yki was shown to produce strong nuclear accumulation, a common feature, coupled with aberrant activity of Yki [47]. Another two residues of Yki are believed to be targets of Wts phosphorylation (Ser111 and Ser250); however, little is known about the underlying mechanisms. As mentioned before, Yki cooperates with many DNA-binding proteins which act as transcription factors, potentiating their function. It is worth noting that some binding partners of Yki are the same kinases that function upstream to it in the Hippo pathway. Thus, through the PY (PPxY)-WW domain interactions, Yki is able to bind to Ex, Wts, and Hpo that sequester Yki at a cytoplasmatic level, independently from its phopshorylated state [48, 49]. Loss of Hippo signaling and consecutive aberrant Yki activation leads to deregulation of some gene class transcriptions. One class includes genes involved in cell survival and proliferation. One of the Yki partners, Scalloped (Sc), a member of TEAD/TEFs family, is responsible for Yki overexpression induced tissue overgrowth [50, 51]. Another partner of Yki in *Drosophila *is Homothorax (Hth) that promotes cell survival and cell proliferation in eye development from eye imaginal discs [52]. Both Sc and Hth are able to bind a Hippo consensus DNA motif, termed Hippo response element (HRE), which is present in many Hippo target genes. Particularly, Sc together with Yki bind to the HRE present in a very well-known target gene, *diap1* [50], an apoptosis inhibitor, as mentioned above. Hth has only little influence on *diap1* transcription. It is very important in regulating the transcription of another Yki target, the growth promoting microRNA gene *bantam.* Other Yki targets in this class are the cell-cycle regulators *cyc* *e*, *e2f1* [4, 53], and *Drosophila* Myc (dMyc) whose expression seems to be positively regulated by Yki [54, 55]. Another important class is made up of components from other signaling pathways, such as ligands for Notch, Wnt, EGFR, and Jak-Stat pathways. In fact, other known Yki partners are believed to be Smad proteins [56]. This interaction appears to potentiate the transcriptional response to BMP/TGF-*β* signaling, addressing a possible crosstalk between Hippo and BMP/TGF-*β* pathway. Finally, a third class of Yki targets consisting of several proteins from its own Hippo cascade, such as Ex, Mer, Kibra, Crb, and Fj. These are downstream transcriptional targets of Yki [9, 17, 20, 57] and define a sort of positive feedback loop which characterizes most signal pathways.

## 3. The Hippo Kinase Signaling in Mammals

### 3.1. YAP and TAZ: Mammalian Effectors of Hippo Pathway

The Hippo pathway is highly conserved in mammalian systems. It was demonstrated that loss of function of mutant flies can be rescued by expressing their respective human counterparts [5, 6]. These data strongly correlate with the importance of Hippo signaling in controlling organ size, tumorigenesis as well as the insurgence of other important diseases in mammals. The ortholog human counterparts of core kinases Hpo and Warts are represented by the pro-apoptotic MST1/2 and LATS1/2 kinases [58, 59] (Figure 2). One ortholog exists for the adaptor protein Sav, termed WW45 or SAV1, and the other two orthologs for Mats are termed MOBKL1A and MOBKL1B (referred to as Mob1). These proteins form a conserved kinase cassette that phosphorylates and inactivates the mammalian Yki homologs YAP and TAZ [25, 47, 60] in response to cell density. This cell density-dependent activation of the Hippo pathway is required in contacting inhibition of cultured mammalian cells [47]. Similar to *Drosophila* Hippo signaling, all the mammalian components of the Hippo pathway clearly show tumor suppression activity. In fact, transgenic overexpression of YAP [61, 62] and liver-specific knockout of *Mst1/2* or *Sav1* [63–66] induce abnormal liver expansion in terms of size, and eventually hepatocellular carcinoma formation (HCC). YAP was initially identified as a 65 kDa binding partner of *c*-Yes from Sudol and collaborators [67]. YAP is a transcriptional co-activator of many transcription factors via its own WW-domain (reviewed in [68]). The TEAD/TEF family of transcription factors, whose homolog is represented by Sc in *Drosophila*, is considered the major partner of both YAP and TAZ in executing their activities within the Hippo pathway. The 4 mammalian TEF/TAED transcription factors are widely expressed and regulate transcription in specific tissues during certain development stages [69]. It was shown that TAED1/TEF2 and YAP share a large number of target genes [51, 70, 71]. In support of this evidence, TEAD1 and TEAD2 double-knockout mice display similar phenotypes to YAP knockouts [69]. Furthermore, ablation of TAED/TEF expression decreases the ability of YAP/TAZ in promoting anchorage independent growth and EMT (epithelial to mesenchymal transition) [51, 71, 72]. Recently Dupont and collaborators have identified YAP and TAZ as the nuclear principal complex of mechanical signals exerted by extracellular matrix (ECM) rigidity and cell shape. This regulation requires Rho GTPase activity and tension of the actomyosin cytoskeleton but is independent from the Hippo/LATS cascade. YAP/TAZ is required for differentiation of mesenchymal stem cells induced by ECM stiffness and for survival of endothelial cells regulated by cell geometry [73].

The exact role of YAP has yet to be defined since it appears to be able to act as an oncogene or as a tumor suppressor depending on the cellular context. YAP1 was shown to bind long forms of p73 and p63, while not to wt p53, thereby potentiating p73- and p63-induced apoptosis [74, 75]. In particular, p73 recapitulates the most well-characterized p53 antitumoral effects, from growth arrest and apoptosis to senescence. YAP imparts transcriptional target specificity to p73 in promoting either growth arrest or apoptosis in response to different stimuli [76–78].

### 3.2. The Complexity of Upstream Regulators: FRMD6, Mer, and Kibra

As mentioned above, the complexity of molecular links between the upstream regulators and the core kinases in mammals has not been clarified either for *Drosophila*. The mammalian genome contains homologs for all the reported upstream regulators of the Hippo pathway. Notably, it encodes more than one paralogue for each *Drosophila* component, thus increasing complexity and the need for further investigation. Two homologs for Kibra, KIBRA/WWC1 and WWC2 and for Expanded, FRMD6 and FRMD1, while only one for Merlin, NF2, were identified. Interestingly, they often differ in protein structure compared to *Drosophila* counterparts. One Ex homolog for FRMD6 does not possess the extended C-terminal portion that is required for growth inhibition activity of Ex and binding to Kibra [20, 79]. No interaction between FRM6 and MST1/2 has been confirmed, in contrast to the described interaction between Ex and Hippo [21]. Also Mer/NF2 is a FERM domain-containing protein and the most investigated. It is a tumor suppressor, whose mutations trigger neurofibromatosis 2, mainly characterized by tumor insurgence in the nervous system [80, 81]. It has a prominent role in growth inhibition triggered by C-adherin-based cell contact. Growth inhibitory action of Mer/NF2 appears to stem from controlling the distribution and signaling of membrane receptors. In fact, in Merlin K/D cells the activation and internalization of the EGF receptor are also maintained in high-cell-density conditions [82]. Furthermore, contrasting data for Mer/NF2 involvement in developing hepatocellular carcinoma (HCC) and tumors of the bile duct were reported. It is worthy to note that in specific *Merlin* ^−/−^ liver an increased proliferation of hepatocytes and of bile ducts was reported, coupled with minor LATS and YAP phopshorylation and increased YAP nuclear export [83]. Conversely, in this context, other authors did not observe any alterations in YAP phosphorylation and localization [84].

### 3.3. The Core Kinases: MST, LATS, and MOB

The ortholog human counterparts of core kinases Hpo and Warts are represented by the proapoptotic MST1/2 and LATS1/2 kinases [58, 59]. MST1/2 are serine-threonine kinases, better known for their ability to initiate apoptosis when overexpressed through a combination of p53- as well as JNK-mediated pathways [85, 86]. Generally, apoptosis induced by different stimuli is coupled with the activation of kinases MST1/2, which result themselves as substrates for caspases 3, 6, and 7 cleavage. This produces highly active catalytic fragments, which are mainly localized in the nucleus, where they exert their proapoptotic function [85–87]. As mentioned above, loss of function of the MST1/2 ortholog Hpo shows a phenotype characterized by a marked overgrowth due to accelerated cell-cycle progression and deregulated apoptosis. Exogenous MST2 expression can successfully rescue this phenotype. MSTs become activated by autophosphorylation in the threonine residues within their activation loop domain. Inhibition of dimerization and autophosphorylation of MST2 exerted by RAF1 was reported [88]. In this latter context, expression of *rassf1a* is able to release MST2 from RAF1 inhibition, thus inducing apoptosis [77]. Moreover, PP2A phosphatase dephosphorylates MST1/2 kinases as shown by two different groups [42, 89]. How autophosphorylation and activation of MST kinases are triggered by unknown extracellular stimuli remain to be elucidated, and okadaic acid treatment or siRNA-mediated knockdown of PP2A promote MST1/2 phopshorylation and activation. Interestingly, Guo and collaborators very recently showed that *rassf1a* activates MST1 and MST2 by preventing their dephosphorylation. Specifically, they observed that *rassf1a* knockdown, which is a frequent phenomenon in human tumors, leads to a dramatic decreased in MST1/2 levels exerted by phosphates. They also observed that restoring *rassf1a* expression and function promotes the formation of active MST1/2 by counteracting the role of phosphates. This is one of the first examples of a tumor suppressor acting as an inhibitor of a specific dephosphorylation pathway.

In the Hippo pathway context, MST substrates include LATS and MOB1. LATS1/2 kinases control cellular homeostasis, negatively regulating cell division cycle 2 (CDC2) and favoring G2/M arrest [90–92]. LATS2 was also reported to induce G1/S arrest [93]. In fact, both overexpressions of LATS1 and 2 dramatically inhibit both cell proliferation and anchorage-independent growth [47, 94] in various cell lines. It is also true that loss of LATS1/2 leads to a broad variety of tumors, such as soft tissue sarcoma and leukemia [95]. In light of these data, these proteins are believed to be strong tumor suppressors. Recent data addressed LATS involvement in tumor suppressive as well as oncogenic pathways, such as p53, RAS, and Akt signaling pathways. Interestingly, LATS2 can bind to MDM2 protein, thus inhibiting its E3 ubiquitin ligase activity to stabilize p53, which in turn favors the transcription of LATS2 [96]. Up until now, YAP and TAZ are the main LATS substrates identified in its kinase activity, but yet they only mediate some of the effects of LATS, thus indicating the existence of other substrates, such as Snail [97], DYRK1A [98], and LATS1 and LATS2 [99]. 

In the Hippo pathway context, LATS activity is supported by MOB1. This protein, which corresponds to the human ortholog of the Mats adaptor protein, binds to and phosphorylates LATS kinases, favoring YAP and TAZ proto-oncogenes phosphorylation and inhibiting their nuclear activity. MOB1 binding to LATS kinases is strongly enhanced upon phosphorylation of MOB1 by MST1/2 kinases [46]. Loss of MOB1 function results in increased cell proliferation and decreased cell death, suggesting that MOB1 functions, as well as the other Hippo pathway components, as a tumor suppressor protein.

## 4. *rassf1a* Signaling into Hippo Pathway 

Due to the absence of enzyme activity, Ras-Association Domain Family (*rassf*) are noncatalytic-proteins. They are often referred to as “scaffold proteins,” which are ubiquitously expressed in normal tissue and described in literature as a strong tumor suppressor family of proteins (reviewed in [100]). The *rassf*s family comprise ten members from *rassf1* to *rassf10*. Among them only *rassf1a* shares the closest homology to *Drosophila* *rassf* (*drassf*) (reviewed in [101]). *rassf1a* exhibits strong tumor suppressor function [102]. Loss of *rassf1a* allele is a frequent occurrence in primary human cancers [103, 104]. Furthermore, hypermethylation of *rassf1a* promoter is very often correlated with oncogenic phenotypes. Concomitantly, the identification of specific point mutations of *rassf1a* impinges on the ability of this protein to inhibit tumor cell growth [105, 106]. About 15% of primary tumors show point mutations of *rassf1a* [107]. Two independent research groups generated *rassf1a* knockout mice [108, 109]. Both these mice showed a phenotype with greatly increased susceptibility to tumor formation. Pursuing the hypothesis that the protein-protein interaction of YAP pattern changes as a consequence of different stimuli, Matallanas and colleagues followed the behavior of *rassf1a* after triggering apoptosis [77]. They showed that *rassf1a* disrupts the inhibitory complex between RAF1 and MST2 and favors the physical association between MST2 and LATS1 concomitantly, therefore, leading to YAP1 phosphorylation and nuclear relocalization where it binds to p73 and potentiates its apoptotic activity (Figure 2). It was also shown that the FAS active receptor induces *rassf1a* to compete with RAF1 in binding to MST2, thus promoting the formation of a LATS1 complex. This results in the translocation of YAP from the cytoplasm to the nucleus. These findings may suggest that the activation of the *rassf1a* complex indirectly diverts LATS1 from phosphorylating YAP, thus making it available for different phosphorylation events. In addition, it is also able to enter into the nucleus where it can activate the transcription of p73 target genes involved in apoptosis. 

It is worthy to note that in 2009, Hamilton and collaborators identified a novel DNA damage pathway that is activated by ATM kinase, involving *rassf1a* and Hippo pathway members [110]. They showed that, upon DNA damage, *rassf1a* becomes phopshorylated by ATM on Ser131. This event seems to be necessary in promoting MST2 binding to *rassf1a*, potentiating MST2 and LATS1 proapoptotic activity leading to p73 stabilization. Thus, this confirms findings observed in previous *in* *vitro* experiments showing that the *rassf1a* peptide containing an ATM putative domain is a substrate for ATM phosphorylation [111, 112]. 

More recently, the interaction, between *rassf1a* and SAV Hippo pathway member [113], was shown to potentiate p73-dependent apoptosis [114]. While this effect does not seem to require direct interaction between *rassf1a* and MST kinases, it was shown to trigger apoptosis via the MST/LATS pathway [77]. It is also true that SAV acts as a scaffold protein connecting MST kinases with LATS kinases [115] and that the expression of exogenous SAV can greatly enhance this proapoptotic signal [113]. Consequently, it is reasonable for authors to speculate the existence of a functional axis involving *rassf1a*-MST-SAV-LATS-YAP in promoting p73-induced apoptosis. Altogether, these findings show a close functional interconnection between *rassf1a*, Hippo, and p53 family tumor suppressor effects. 

RASFF1A functions as a negative regulator of cardiomyocyte hypertrophy [116]. The latter displays an enlargement in size of cardiomyocytes, which is very often associated with heart failure [117]. It was proposed that a large number of protooncogenes, which are expressed in the heart, could possibly mediate this aberrant process [118]. *rassf1a* exon1*α* knockout mice exhibit normal cardiac morphology at 12 weeks of age. Notably, the application of a pressure overloaded the transverse aortic constriction causing massive cardiac hypertrophy, among the severest reactions ever to be reported [116]. This may suggest that *rassf1a* plays a role in contrasting overproliferation of cardiomyocytes. Interestingly, the authors observed that *rassf1a* in this cellular system greatly opposes the RAS-RAF1-ERK1/2 signal pathway. Not long ago, it was proposed that the activation of RAF by RAS requires a complex regulation of many adaptor molecules including the involvement of CNK1 (connector enhancer of kinase suppressor of RAS). This protein is able to form a complex with *rassf1a*, increasing *rassf1a*-induced cell death [119]. In light of these data authors speculated about a possible imbalance in the ratio of the components of the scaffold complex required for RAS signal transmission. CNK1 was also found to interact with MST1 and MST2, requiring MST kinases to induce apoptosis. Deleting the MST1 segment that mediates binding to *rassf1a* also eliminates the physical association between MST1 and CNK1. To sum up, CNK1 binds to *rassf1a* and promotes apoptosis through a pathway that requires *rassf1a* and MST kinases [119]. This mechanism may be the underlying factor behind *rassf1a*'s action in preventing cardiomyocytes hypertrophy. Supporting this, Del Re and collaborators showed that *rassf1a* is an endogenous activator of MST1 in the heart. They also found that in cardiac fibroblasts the *rassf1a*/MST1 pathway negatively regulates TNF-*α* that is believed to be a key mediator of hypertrophy and consecutive cardiac dysfunction [120]. Altogether, these findings highlight the importance of a crosstalk between *rassf1a* and components of the human Hippo pathway in preventing cardiac dysfunction due to aberrant overproliferation of cardiomyocytes. Of note, other Hippo pathway members were shown to be involved in heart development and size, such as YAP [121], Dch1-FAT [122], LATS2 [123], and SAV [124]. 

## 5. *rassf5* and *rassf6*

Other *rassf* family members were involved in modulating the activity of Hippo pathway components. The first RAS interactor discovered within this family was *rassf5* [125], often called Novel Ras Effector 1 (NORE1). This isoform that shares up to 60% homology with *rassf1*, is the most common isoform. As for many *rassf*s, it was demonstrated to be a centrosomal protein that can bind to the microtubule scaffold structure. This event appears to be required for growth inhibition and consequently tumor suppression activity, which is achieved through the inhibition of ERK signaling [126]. Furthermore, it has been reported that active RAS binds to *rassf5*-MST1 complex thereby conferring the role of the RAS effector complex in mediating the proapoptotic function of KiRASG12V [127]. RASFF5 and the MST1 pro-apoptotic kinase are involved in a physical interaction, thus forming an active complex where RAS interacts upon serum stimulation consequently leading to its pro-apoptotic function. Furthermore, the interaction of *rassf1a* and NORE1 with MST1 appears to be controversial. In fact, an inhibition of MST kinases activity by coexpression with the complex NORE1-*rassf1a* in excess was reported [128]. At the same time, by *in vivo * experiments, overexpression of *rassf1a* together with MST2 was shown to increase kinase activity of MST2 consequently potentiating its pro-apoptotic effect [77, 113, 129]. 

In 2009, Ikeda and collaborators showed that another *rassf* member, *rassf*6, can bind to MST2 kinase. This protein is known to induce apoptosis [130, 131]. When *rassf*6 is bound to MST2, *rassf*6 inhibits MST2 activity, thus, inhibiting its role in the Hippo pathway. Conversely, the release of MST2 from *rassf*6 causes apoptosis in a WW45-dependent manner (*Drosophila* SAV). Therefore, *rassf*6 impinges the Hippo proapoptotic pathway by inhibiting MST2, but it is *per se* able to induce apoptosis through a parallel Hippo mechanism. In fact, MST2 is responsible for apoptosis induced through Hippo signaling and through a *rassf6*-WW45-mediated pathway [131].

## 6. Concluding Remarks and Future Perspectives

In conclusion, the Hippo pathway is a signaling pathway that regulates cell proliferation and cell death. It is a kinase cascade that phosphorylates and negatively regulates transcription by transcriptional coactivators. As summarized above, the loss of function of the Hippo pathway triggers tumorigenesis. Accordingly, the downregulation of the Hippo pathway is frequently observed in human cancers. Aberrant activation of Hippo downstream executors, YAP1 and TAZ, induce epithelial-mesenchymal transition and the expression of stem-cell markers in cancer cells. Quite recently, the Hippo and the *rassf* pathways have emerged to be closely linked. The tumor suppressor *rassf* proteins were shown to induce cell-cycle arrest and apoptosis. Stimuli activating the Hippo pathway simultaneously induce *rassf*-dependent biological events. Thereby, the Hippo and *rassf* pathways cooperate in preventing tumorigenesis. Reintegration of the Hippo pathway and *rassf* functions should be implemented in cancer therapy. However, it is also true that if this cross-talk results disproportionate, the consequence will be excessive apoptosis and consecutive organ dysfunction. In such cases, the involvement of the Hippo/*rassf* inhibitors will be useful. The relationship between the Hippo and *rassf* pathways is probably not restricted to cancer biology since many of the Hippo components also regulate adipogenesis, osteogenesis, and myogenesis. As discussed above, a growing body of evidence shows that this relationship between *rassf* and the Hippo pathways also occurs in cardiac tissue inhibiting cardiac hypertrophy and playing a critical role in preventing heart failure. Based on what has been described and in light of the synergistic effects observed on the interaction within *rassf* and components of Hippo signaling in preventing defects of proper biological development such as insurgence of many human diseases, much more work is needed to further investigate the importance of this physiological relationship.

## References

[1] Pan D (2010). The hippo signaling pathway in development and cancer. *Developmental Cell*.

[2] Zhao B, Tumaneng K, Guan KL (2011). The hippo pathway in organ size control, tissue regeneration and stem cell self-renewal. *Nature Cell Biology*.

[3] Justice RW, Zilian O, Woods DF, Noll M, Bryant PJ (1995). The *Drosophila* tumor suppressor gene warts encodes a homolog of human myotonic dystrophy kinase and is required for the control of cell shape and proliferation. *Genes and Development*.

[4] Tapon N, Harvey KF, Bell DW (2002). salvador promotes both cell cycle exit and apoptosis in *Drosophila* and is mutated in human cancer cell lines. *Cell*.

[5] Wu S, Huang J, Dong J, Pan D (2003). hippo encodes a Ste-20 family protein kinase that restricts cell proliferation and promotes apoptosis in conjunction with salvador and warts. *Cell*.

[6] Lai ZC, Wei X, Shimizu T (2005). Control of cell proliferation and apoptosis by mob as tumor suppressor, mats. *Cell*.

[7] Boedigheimer M, Bryant P, Laughon A (1993). Expanded, a negative regulator of cell proliferation in *Drosophila*, shows homology to the NF2 tumor suppressor. *Mechanisms of Development*.

[8] LaJeunesse DR, McCartney BM, Fehon RG (1998). Structural analysis of *Drosophila* Merlin reveals functional domains important for growth control and subcellular localization. *Journal of Cell Biology*.

[9] Hamaratoglu F, Willecke M, Kango-Singh M (2006). The tumour-suppressor genes NF2/Merlin and expanded act through hippo signalling to regulate cell proliferation and apoptosis. *Nature Cell Biology*.

[10] Maitra S, Kulikauskas RM, Gavilan H, Fehon RG (2006). The tumor suppressors Merlin and expanded function cooperatively to modulate receptor endocytosis and signaling. *Current Biology*.

[11] Mahoney PA, Weber U, Onofrechuk P, Biessmann H, Bryant PJ, Goodman CS (1991). The fat tumor suppressor gene in *Drosophila* encodes a novel member of the cadherin gene superfamily. *Cell*.

[12] Bennett FC, Harvey KF (2006). Fat cadherin modulates organ size in *Drosophila* via the Salvador/Warts/hippo signaling pathway. *Current Biology*.

[13] Silva E, Tsatskis Y, Gardano L, Tapon N, McNeill H (2006). The tumor-suppressor gene fat controls tissue growth upstream of expanded in the hippo signaling pathway. *Current Biology*.

[14] Willecke M, Hamaratoglu F, Kango-Singh M (2006). The fat cadherin acts through the hippo tumor-suppressor pathway to regulate tissue size. *Current Biology*.

[15] Matakatsu H, Blair SS (2004). Interactions between Fat and Dachsous and the regulation of planar cell polarity in the *Drosophila* wing. *Development*.

[16] Matakatsu H, Blair SS (2006). Separating the adhesive and signaling functions of the Fat and Dachsous protocadherins. *Development*.

[17] Cho E, Feng Y, Rauskolb C, Maitra S, Fehon R, Irvine KD (2006). Delineation of a Fat tumor suppressor pathway. *Nature Genetics*.

[18] Feng Y, Irvine KD (2009). Processing and phosphorylation of the Fat receptor. *Proceedings of the National Academy of Sciences of the United States of America*.

[19] Baumgartner R, Poernbacher I, Buser N, Hafen E, Stocker H (2010). The WW domain protein kibra acts upstream of hippo in *Drosophila*. *Developmental Cell*.

[20] Genevet A, Wehr MC, Brain R, Thompson BJ, Tapon N (2010). Kibra is a regulator of the Salvador/Warts/hippo signaling network. *Developmental Cell*.

[21] Yu J, Zheng Y, Dong J, Klusza S, Deng WM, Pan D (2010). Kibra functions as a tumor suppressor protein that regulates hippo signaling in conjunction with Merlin and Expanded. *Developmental Cell*.

[22] Chen CL, Gajewski KM, Hamaratoglu F (2010). The apical-basal cell polarity determinant Crumbs regulates hippo signaling in *Drosophila*. *Proceedings of the National Academy of Sciences of the United States of America*.

[23] Ling C, Zheng Y, Yin F (2010). The apical transmembrane protein Crumbs functions as a tumor suppressor that regulates hippo signaling by binding to expanded. *Proceedings of the National Academy of Sciences of the United States of America*.

[24] Robinson BS, Huang J, Hong Y, Moberg KH (2010). Crumbs regulates Salvador/Warts/hippo signaling in *Drosophila* via the FERM-domain protein expanded. *Current Biology*.

[25] Huang J, Wu S, Barrera J, Matthews K, Pan D (2005). The hippo signaling pathway coordinately regulates cell proliferation and apoptosis by inactivating Yorkie, the *Drosophila* homolog of YAP. *Cell*.

[26] Sudol M, Chen HI, Bougeret C, Einbond A, Bork P (1995). Characterization of a novel protein-binding module—the WW domain. *FEBS Letters*.

[27] Sudol M (2010). Newcomers to the WW domain-mediated network of the hippo tumor suppressor pathway. *Genes and Cancer*.

[28] Sudol M, Harvey KF (2010). Modularity in the hippo signaling pathway. *Trends in Biochemical Sciences*.

[29] Tepass U, Theres C, Knust E (1990). *Crumbs* encodes an EGF-like protein expressed on apical membranes of *Drosophila* epithelial cells and required for organization of epithelia. *Cell*.

[30] Yang CH, Axelrod JD, Simon MA (2002). Regulation of Frizzled by Fat-like cadherins during planar polarity signaling in the *Drosophila* compound eye. *Cell*.

[31] Casal J, Lawrence PA, Struhl G (2006). Two separate molecular systems, Dachsous/Fat and Starry night/Frizzled, act independently to confer planar star polarity. *Development*.

[32] Feng Y, Irvine KD (2007). Fat and expanded act in parallel to regulate growth through Warts. *Proceedings of the National Academy of Sciences of the United States of America*.

[33] Polesello C, Tapon N (2007). Salvador-Warts-hippo signaling promotes *Drosophila* posterior follicle cell maturation downstream of notch. *Current Biology*.

[34] Reddy BVVG, Rauskolb C, Irvine KD (2010). Influence of Fat-hippo and Notch signaling on the proliferation and differentiation of *Drosophila* optic neuroepithelia. *Development*.

[35] Rogulja D, Rauskolb C, Irvine KD (2008). Morphogen control of wing growth through the Fat signaling pathway. *Developmental Cell*.

[36] Willecke M, Hamaratoglu F, Sansores-Garcia L, Tao C, Halder G (2008). Boundaries of Dachsous Cadherin activity modulate the hippo signaling pathway to induce cell proliferation. *Proceedings of the National Academy of Sciences of the United States of America*.

[37] Ishikawa HO, Takeuchi H, Haltiwanger RS, Irvine KD (2008). Four-jointed is a Golgi kinase that phosphorylates a subset of cadherin domains. *Science*.

[38] Matakatsu H, Blair SS (2008). The DHHC palmitoyltransferase approximated regulates Fat signaling and Dachs localization and activity. *Current Biology*.

[39] Rauskolb C, Pan G, Reddy BVVG, Oh H, Irvine KD (2011). Zyxin links fat signaling to the hippo pathway. *PLoS Biology*.

[40] Harvey K, Tapon N (2007). The Salvador-Warts-hippo pathway—an emerging tumour-suppressor network. *Nature Reviews Cancer*.

[41] Polesello C, Huelsmann S, Brown N, Tapon N (2006). The *Drosophilarassf* homolog antagonizes the hippo pathway. *Current Biology*.

[42] Ribeiro PS, Josué F, Wepf A (2010). Combined functional genomic and proteomic approaches identify a PP2A complex as a negative regulator of hippo signaling. *Molecular Cell*.

[43] Grzeschik NA, Parsons LM, Allott ML, Harvey KF, Richardson HE (2010). Lgl, aPKC, and Crumbs regulate the Salvador/Warts/hippo pathway through two distinct mechanisms. *Current Biology*.

[44] Dong S, Kang S, Gu TL (2007). 14-3-3 Integrates prosurvival signals mediated by the AKT and MAPK pathways in ZNF198-FGFR1-transformed hematopoietic cells. *Blood*.

[45] Oh H, Irvine KD (2008). In vivo regulation of Yorkie phosphorylation and localization. *Development*.

[46] Praskova M, Xia F, Avruch J (2008). MOBKL1A/MOBKL1B Phosphorylation by MST1 and MST2 Inhibits Cell Proliferation. *Current Biology*.

[47] Zhao B, Wei X, Li W (2007). Inactivation of YAP oncoprotein by the hippo pathway is involved in cell contact inhibition and tissue growth control. *Genes and Development*.

[48] Badouel C, Gardano L, Amin N (2009). The FERM-domain protein Expanded regulates hippo pathway activity via direct interactions with the transcriptional activator Yorkie. *Developmental Cell*.

[49] Oh H, Reddy BVVG, Irvine KD (2009). Phosphorylation-independent repression of Yorkie in Fat-hippo signaling. *Developmental Biology*.

[50] Wu S, Liu Y, Zheng Y, Dong J, Pan D (2008). The TEAD/TEF family protein Scalloped mediates transcriptional output of the hippo growth-regulatory pathway. *Developmental cell*.

[51] Zhang L, Ren F, Zhang Q, Chen Y, Wang B, Jiang J (2008). The TEAD/TEF family of transcription factor Scalloped mediates hippo signaling in organ size control. *Developmental cell*.

[52] Peng HW, Slattery M, Mann RS (2009). Transcription factor choice in the hippo signaling pathway: homothorax and yorkie regulation of the microRNA bantam in the progenitor domain of the *Drosophila* eye imaginal disc. *Genes and Development*.

[53] Goulev Y, Fauny JD, Gonzalez-Marti B, Flagiello D, Silber J, Zider A (2008). SCALLOPED interacts with YORKIE, the nuclear effector of the hippo tumor-suppressor pathway in *Drosophila*. *Current Biology*.

[54] Neto-Silva RM, de Beco S, Johnston LA (2010). Evidence for a growth-stabilizing regulatory feedback mechanism between Myc and Yorkie, the *Drosophila* homolog of Yap. *Developmental Cell*.

[55] Ziosi M, Baena-López LA, Grifoni D (2010). dMyc functions downstream of yorkie to promote the supercompetitive behavior of hippo pathway mutant cells. *PLoS Genetics*.

[56] Alarcón C, Zaromytidou AI, Xi Q (2009). Nuclear CDKs drive smad transcriptional activation and turnover in BMP and TGF-*β* pathways. *Cell*.

[57] Genevet A, Polesello C, Blight K (2009). The hippo pathway regulates apical-domain size independently of its growth-control function. *Journal of Cell Science*.

[58] Hergovich A, Hemmings BA (2009). Mammalian NDR/LATS protein kinases in hippo tumor suppressor signaling. *BioFactors*.

[59] Radu M, Chernoff J (2009). The DeMSTification of mammalian Ste20 kinases. *Current Biology*.

[60] Lei QY, Zhang H, Zhao B (2008). TAZ promotes cell proliferation and epithelial-mesenchymal transition and is inhibited by the hippo pathway. *Molecular and Cellular Biology*.

[61] Camargo FD, Gokhale S, Johnnidis JB (2007). YAP1 Increases Organ Size and Expands Undifferentiated Progenitor Cells. *Current Biology*.

[62] Dong J, Feldmann G, Huang J (2007). Elucidation of a Universal Size-Control Mechanism in *Drosophila* and Mammals. *Cell*.

[63] Zhou D, Conrad C, Xia F (2009). Mst1 and Mst2 Maintain Hepatocyte Quiescence andSuppress Hepatocellular Carcinoma Development through Inactivation of the Yap1 Oncogene. *Cancer Cell*.

[64] Lee KP, Lee JH, Kim TS (2010). The hippo-Salvador pathway restrains hepatic oval cell proliferation, liver size, and liver tumorigenesis. *Proceedings of the National Academy of Sciences of the United States of America*.

[65] Lu L, Li Y, Kim SM (2010). hippo signaling is a potent in vivo growth and tumor suppressor pathway in the mammalian liver. *Proceedings of the National Academy of Sciences of the United States of America*.

[66] Song H, Mak KK, Topol L (2010). Mammalian Mst1 and Mst2 kinases play essential roles in organ size control and tumor suppression. *Proceedings of the National Academy of Sciences of the United States of America*.

[67] Sudol M, Bork P, Einbond A (1995). Characterization of the mammalian YAP (Yes-associated protein) gene and its role in defining a novel protein module, the WW domain. *Journal of Biological Chemistry*.

[68] Bertini E, Oka T, Sudol M, Strano S, Blandino G (2009). At the crossroad between transformation and tumor suppression. *Cell Cycle*.

[69] Sawada A, Kiyonari H, Ukita K, Nishioka N, Imuta Y, Sasaki H (2008). Redundant roles of Tead1 and Tead2 in notochord development and the regulation of cell proliferation and survival. *Molecular and Cellular Biology*.

[70] Ota M, Sasaki H (2008). Mammalian Tead proteins regulate cell proliferation and contact inhibition as transcriptional mediators of hippo signaling. *Development*.

[71] Zhao B, Ye X, Yu J (2008). TEAD mediates YAP-dependent gene induction and growth control. *Genes and Development*.

[72] Chan SW, Lim CJ, Loo LS, Chong YF, Huang C, Hong W (2009). TEADs mediate nuclear retention of TAZ to promote oncogenic transformation. *Journal of Biological Chemistry*.

[73] Dupont S, Morsut L, Aragona M (2011). Role of YAP/TAZ in mechanotransduction. *Nature*.

[74] Sudol M, Hunter T (2000). New wrinkles for an old domain. *Cell*.

[75] Strano S, Munarriz E, Rossi M (2001). Physical Interaction with Yes-associated Protein Enhances p73 Transcriptional Activity. *Journal of Biological Chemistry*.

[76] Strano S, Monti O, Pediconi N (2005). The transcriptional coactivator yes-associated protein drives p73 gene-target specificity in response to DNA damage. *Molecular Cell*.

[77] Matallanas D, Romano D, Yee K (2007). *rassf1a* Elicits Apoptosis through an MST2 Pathway Directing Proapoptotic Transcription by the p73 Tumor Suppressor Protein. *Molecular Cell*.

[78] Lapi E, Di Agostino S, Donzelli S (2008). PML, YAP, and p73 Are Components of a Proapoptotic Autoregulatory Feedback Loop. *Molecular Cell*.

[79] Boedigheimer MJ, Nguyen KP, Bryant PJ (1997). expanded functions in the apical cell domain to regulate the growth rate of imaginal discs. *Developmental Genetics*.

[80] Rouleau GA, Merel P, Lutchman M (1993). Alteration in a new gene encoding a putative membrane-organizing protein causes neuro-fibromatosis type 2. *Nature*.

[81] Trofatter (1993). Erratum: A novel moesin-, ezrin-, and radixin-like gene is a candidate for the neurofibromatosis 2 tumor suppressor. *Cell*.

[82] Curto M, Cole BK, Lallemand D, Liu CH, McClatchey AI (2007). Contact-dependent inhibition of EGFR signaling by Nf2/Merlin. *Journal of Cell Biology*.

[83] Zhang N, Bai H, David KK (2010). The Merlin/NF2 Tumor Suppressor Functions through the YAP Oncoprotein to Regulate Tissue Homeostasis in Mammals. *Developmental Cell*.

[84] Benhamouche S, Curto M, Saotome I (2010). Nf2/Merlin controls progenitor homeostasis and tumorigenesis in the liver. *Genes and Development*.

[85] Lin Y, Khokhlatchev A, Figeys D, Avruch J (2002). Death-associated protein 4 binds MST1 and augments MST1-induced apoptosis. *Journal of Biological Chemistry*.

[86] Ura S, Nishina H, Gotoh Y, Katada T (2007). Activation of the c-Jun N-terminal kinase pathway by MST1 is essential and sufficient for the induction of chromatin condensation during apoptosis. *Molecular and Cellular Biology*.

[87] Anand R, Kim AY, Brent M, Marmorstein R (2008). Biochemical analysis of MST1 kinase: Elucidation of a C-terminal regulatory region. *Biochemistry*.

[88] O’Neill E, Rushworth L, Baccarini M, Kolch W (2004). Role of the kinase MST2 in suppression of apoptosis by the proto-oncogene product Raf-1. *Science*.

[89] Guo C, Zhang X, Pfeifer GP (2011). The tumor suppressor *rassf1a* prevents dephosphorylation of the mammalian STE20-like kinases MST1 and MST2. *Journal of Biological Chemistry*.

[90] Yang X, Li DM, Chen W, Xu T (2001). Human homologue of *Drosophila* lats, LATS1, negatively regulate growth by inducing G2/M arrest or apoptosis. *Oncogene*.

[91] Xia H, Qi H, Li Y (2002). LATS1 tumor suppressor regulates G2/M transition and apoptosis. *Oncogene*.

[92] Yabuta N, Okada N, Ito A (2007). LATS2 is an essential mitotic regulator required for the coordination of cell division. *Journal of Biological Chemistry*.

[93] Li Y, Pei J, Xia H, Ke H, Wang H, Tao W (2003). LATS2, a putative tumor suppressor, inhibits G1/S transition. *Oncogene*.

[94] Aylon Y, Yabuta N, Besserglick H (2009). Silencing of the LATS2 tumor suppressor overrides a p53-dependent oncogenic stress checkpoint and enables mutant H-Ras-driven cell transformation. *Oncogene*.

[95] St John MAR, Tao W, Fei X (1999). Mice deficient of Lats1 develop soft-tissue sarcomas, ovarian tumours and pituitary dysfunction. *Nature Genetics*.

[96] Aylon Y, Michael D, Shmueli A, Yabuta N, Nojima H, Oren M (2006). A positive feedback loop between the p53 and LATS2 tumor suppressors prevents tetraploidization. *Genes and Development*.

[97] Zhang K, Rodriguez-Aznar E, Yabuta N (2012). LATS2 kinase potentiates Snail1 activity by promoting nuclear retention upon phosphorylation. *EMBO Journal*.

[98] Tschöp K, Conery AR, Litovchick L (2011). A kinase shRNA screen links LATS2 and the pRB tumor suppressor. *Genes and Development*.

[99] Hori T, Takaori-Kondo A, Kamikubo Y, Uchiyama T (2000). Molecular cloning of a novel human protein kinase, kpm, that is homologous to warts/lats, a *Drosophila* tumor suppressor. *Oncogene*.

[100] van der Weyden L, Adams DJ (2007). The Ras-association domain family (RASSF) members and their role in human tumourigenesis. *Biochimica et Biophysica Acta - Reviews on Cancer*.

[101] Gordon M, Baksh S (2011). *rassf1a*: Not a prototypical Ras effector. *Small GTPases*.

[102] Baksh S, Tommasi S, Fenton S (2005). The tumor suppressor *rassf1a* and MAP-1 link death receptor signaling to bax conformational change and cell death. *Molecular Cell*.

[103] Dammann R, Li C, Yoon JH, Chin PL, Bates S, Pfeifer GP (2000). Epigenetic inactivation of a RAS association domain family protein from the lung tumour suppressor locus 3p21.3. *Nature Genetics*.

[104] Burbee DG, Forgacs E, Zöchbauer-Müller S (2001). Epigenetic inactivation of *rassf1a* in lung and breast cancers and malignant phenotype suppression. *Journal of the National Cancer Institute*.

[105] Kuzmin I, Gillespie JW, Protopopov A (2002). The *rassf1a* tumor suppressor gene is inactivated in prostate tumors and suppresses growth of prostate carcinoma cells. *Cancer Research*.

[106] Shivakumar L, Minna J, Sakamaki T, Pestell R, White MA (2002). The *rassf1a* tumor suppressor blocks cell cycle progression and inhibits cyclin D1 accumulation. *Molecular and Cellular Biology*.

[107] Pan ZG, Kashuba VI, Liu XQ (2005). High frequency somatic mutations in *rassf1a* in nasopharyngeal carcinoma. *Cancer Biology and Therapy*.

[108] Tommasi S, Dammann R, Zhang Z (2005). Tumor susceptibility of *rassf1a* knockout mice. *Cancer Research*.

[109] van der Weyden L, Tachibana KK, Gonzalez MA (2005). The *rassf1a* isoform of *rassf1* promotes microtubule stability and suppresses tumorigenesis. *Molecular and Cellular Biology*.

[110] Hamilton G, Yee KS, Scrace S, O’Neill E (2009). ATM Regulates a *rassf1a*-Dependent DNA Damage Response. *Current Biology*.

[111] Kim ST, Lim DS, Canman CE, Kastan MB (1999). Substrate specificities and identification of putative substrates of ATM kinase family members. *Journal of Biological Chemistry*.

[112] O’Neill T, Dwyer AJ, Ziv Y (2000). Utilization of oriented peptide libraries to identify substrate motifs selected by ATM. *Journal of Biological Chemistry*.

[113] Guo C, Tommasi S, Liu L, Yee JK, Dammann R, Pfeifer G (2007). *rassf1a* Is Part of a Complex Similar to the *Drosophila* hippo/Salvador/Lats Tumor-Suppressor Network. *Current Biology*.

[114] Donninger H, Allen N, Henson A (2011). Salvador protein is a tumor suppressor effector of *rassf1a* with hippo pathway-independent functions. *Journal of Biological Chemistry*.

[115] Saucedo LJ, Edgar BA (2007). Filling out the hippo pathway. *Nature Reviews Molecular Cell Biology*.

[116] Oceandy D, Pickard A, Prehar S (2009). Tumor suppressor ras-association domain family 1 Isoform A Is a novel regulator of cardiac hypertrophy. *Circulation*.

[117] Dorn GW, Robbins J, Sugden PH (2003). Phenotyping hypertrophy: Eschew obfuscation. *Circulation Research*.

[118] Marban E, Koretsune Y (1990). Cell calcium, oncogenes, and hypertrophy. *Hypertension*.

[119] Rabizadeh S, Xavier RJ, Ishiguro K (2004). The scaffold protein CNK1 interacts with the tumor suppressor *rassf1a* and augments *rassf1a*-induced cell death. *Journal of Biological Chemistry*.

[120] Del Re DP, Matsuda T, Zhai P (2010). Proapoptotic *rassf1a*/Mst1 signaling in cardiac fibroblasts is protective against pressure overload in mice. *Journal of Clinical Investigation*.

[121] von Gise A, Lin Z, Schlegelmilch K (2012). YAP1, the nuclear target of hippo signaling, stimulates heart growth through cardiomyocyte proliferation but not hypertrophy. *Proceedings of the National Academy of Sciences of the United States of America*.

[122] Mao Y, Mulvaney J, Zakaria S (2011). Characterization of a Dchs1 mutant mouse reveals requirements for Dchs1-Fat4 signaling during mammalian development. *Development*.

[123] Matsui Y, Nakano N, Shao D (2008). LATS2 is a negative regulator of myocyte size in the heart. *Circulation Research*.

[124] Heallen T, Zhang M, Wang J (2011). hippo pathway inhibits wnt signaling to restrain cardiomyocyte proliferation and heart size. *Science*.

[125] Vavvas D, Li X, Avruch J, Zhang XF (1998). Identification of Nore1 as a potential Ras effector. *Journal of Biological Chemistry*.

[126] Moshnikova A, Frye J, Shay JW, Minna JD, Khokhlatchev AV (2006). The growth and tumor suppressor NORE1A is a cytoskeletal protein that suppresses growth by inhibition of the ERK pathway. *Journal of Biological Chemistry*.

[127] Khokhlatchev A, Rabizadeh S, Xavier R (2002). Identification of a novel Ras-regulated proapoptotic pathway. *Current Biology*.

[128] Praskova M, Khoklatchev A, Ortiz-Vega S, Avruch J (2004). Regulation of the MST1 kinase by autophosphorylation, by the growth inhibitory proteins, *rassf1* and NORE1, and by Ras. *Biochemical Journal*.

[129] Oh HJ, Lee KK, Song SJ (2006). Role of the tumor suppressor *rassf1a* in Mst1-mediated apoptosis. *Cancer Research*.

[130] Allen NPC, Donninger H, Vos MD (2007). *rassf6* is a novel member of the RASSF family of tumor suppressors. *Oncogene*.

[131] Ikeda M, Kawata A, Nishikawa M (2009). hippo pathway-dependent and-independent roles of *rassf6*. *Science Signaling*.

